# Evaluation of the impact of pre-operative stereotactic radiotherapy on the acute changes in histopathologic and immune marker profiles of brain metastases

**DOI:** 10.1038/s41598-022-08507-3

**Published:** 2022-03-16

**Authors:** Rupesh Kotecha, Raees Tonse, Miguel A. Ramirez Menendez, Andre Williams, Zuanel Diaz, Martin C. Tom, Matthew D. Hall, Minesh P. Mehta, Reinier Alvarez, Vitaly Siomin, Yazmin Odia, Manmeet S. Ahluwalia, Michael W. McDermott

**Affiliations:** 1 Department of Radiation Oncology, Miami Cancer Institute, Baptist Health South Florida, Office 1R203, Miami, FL 33176 USA; 2 Herbert Wertheim College of Medicine, Florida International University, Miami, FL USA; 3 Office of Clinical Research, Miami Cancer Institute, Baptist Health South Florida, Miami, FL USA; 4 Department of Neurosurgery, Miami Neuroscience Institute, Baptist Health South Florida, Miami, FL USA; 5 Department of Neuro-Oncology, Miami Cancer Institute, Baptist Health South Florida, Miami, FL USA; 6 Department of Medical Oncology, Miami Cancer Institute, Baptist Health South Florida, Miami, FL USA

**Keywords:** Cancer, Oncology

## Abstract

The unique acute effects of the large fractional doses that characterize stereotactic radiosurgery (SRS) or radiotherapy (SRT), specifically in terms of antitumor immune cellular processes, vascular damage, tumor necrosis, and apoptosis on brain metastasis have yet to be empirically demonstrated. The objective of this study is to provide the first in-human evaluation of the acute biological effects of SRS/SRT in resected brain metastasis. Tumor samples from patients who underwent dose-escalated preoperative SRT followed by resection with available non-irradiated primary tumor tissues were retrieved from our institutional biorepository. All primary tumors and irradiated metastases were evaluated for the following parameters: tumor necrosis, T-cells, natural killer cells, vessel density, vascular endothelial growth factor, and apoptotic factors. Twenty-two patients with irradiated and resected brain metastases and paired non-irradiated primary tumor samples met inclusion criteria. Patients underwent a median preoperative SRT dose of 18 Gy (Range: 15–20 Gy) in 1 fraction, with 3 patients receiving 27–30 Gy in 3–5 fractions, followed by resection within median interval of 67.8 h (R: 18.25–160.61 h). The rate of necrosis was significantly higher in irradiated brain metastases than non-irradiated primary tumors (*p* < 0.001). Decreases in all immunomodulatory cell populations were found in irradiated metastases compared to primary tumors: CD3 + (*p* = 0.003), CD4 + (*p* = 0.01), and CD8 + (*p* = 0.01). Pre-operative SRT is associated with acute effects such as increased tumor necrosis and differences in expression of immunomodulatory factors, an effect that does not appear to be time dependent, within the limited intervals explored within the context of this analysis.

## Introduction

Approximately 20–40% of patients with solid tumors will develop brain metastasis at some point during their disease course, with lung, breast, melanoma, colorectal, and renal cell carcinomas accounting for the vast majority of primary tumors^1^. Amongst the variety of treatment options for medically-operable patients with resectable brain metastasis, surgery is typically performed for those without a known primary site of disease^2^, in the setting of a solitary brain metastasis^3^, or in those with large symptomatic lesions with associated mass effect^4^. Several radiotherapy options exist for patients with resected brain metastasis, including post-operative whole-brain radiotherapy (WBRT)^5^, post-operative stereotactic radiosurgery (SRS)^6^, intraoperative radiotherapy^7^, and brachytherapy^8^. Recent comparative analyses have supported an alternative approach with pre-operative SRS with apparently more favorable rates of local control, radiation necrosis, and leptomeningeal dissemination^9,10^. Pre-operative SRS series have typically used prescription doses with a 20% dose-reduction from the standard Radiation Therapy Oncology Group (RTOG) 90–05 dosing schema^11^, without additional microscopic margin additions, and surgery planned approximately 48 h after resection^12^. Clinical experience with pre-operative SRS is gaining momentum. However, our understanding of the biology of stereotactic radiotherapy (SRT) on brain metastasis and the effect of high fractional doses on immunomodulatory cell populations, endothelial cells and vascular networks, and DNA integrity is limited to preclinical studies^13^ and anecdotal legacy reports^14^, which have largely relied on obtaining tissue at much later time points. Such reports suggest the presence of delayed histopathologic changes following SRS/SRT, but limited information exists regarding the occurrence of acute post-SRS/SRT effects.

Our group has previously described metabolic changes occurring acutely after SRS^15^. In our prior study, four patients, two with malignant gliomas and two with brain metastases, were treated with SRS to 15 and 27.5 Gy to the 80% isodose line covering the contrast-enhancing tumor margin. Patients underwent a sequence of three Positron Emission Tomographic (PET) scans using [18F]-fluorodeoxyglucose (PET-FDG): a baseline scan the day before treatment, and follow-up scans 1 and 7 days after treatment. Ratios between the maximal tumor regional cerebral metabolic rate for glucose and the contralateral remote white matter were calculated. Compared to baseline, ratios increased acutely post-SRS by 25% to 42%, on the 1-day scan, then decreased to between 10% above and 12% below the baseline value 7 days post-SRS. These reports underscore the presence of acute physiologic/metabolic effects, which have not been correlated with histopathologic changes.

The objective of this study is to provide the first in-human evaluation of the acute biological effects of dose-escalated pre-operative SRT for resectable brain metastasis, with significant focus on immune cell population alterations, which may have clinical relevance in an era of increasing utilization of immune checkpoint inhibitors. We hypothesized that metastasis treated with SRT would exhibit differences in tissue parameters associated with antitumor immune cellular processes, vascular damage, tumor necrosis, and apoptosis compared to non-irradiated primary tumors. Given the variability in timing between SRT and surgery in our studied patient population, we also hypothesized that some of these parameters might demonstrate dose or timing dependency.

## Methods

### Patients

Patients who underwent pre-operative SRT followed by resection of brain metastasis were queried from an institutional registry (IRB# 1672008). The study was approved by the Miami Cancer Institute Institutional Review Board, and informed consent was obtained from every patient. All patients were treated with a previously established dose-escalated pre-operative SRS paradigm to the gross tumor volume with no additional clinical target volume or planning target volume expansions^16^. Only patients who also had non-irradiated primary tumor tissue samples available for comparative analyses were eligible for this particular study. Patient information, including primary tumor histology, size, volume, and location as well as treatment details, including prescription dose, number of fractions, and dose per fraction were abstracted from the electronic medical record. The start and end timing of SRS/SRT were extracted from the radiation oncology treatment database, and surgical details, including the time of surgery, were collected from the operative reports. Local failure was defined using the Response Assessment in Neuro-Oncology Brain Metastases (RANO-BM) criteria^17^ with recurrence identified as enhancing tumor apart from post-surgical changes and confirmed by multi-disciplinary peer review. All the methods adhered to relevant ethical guidelines for handling human data.

### Tissue analysis

All non-irradiated primary tumors and paired irradiated brain metastases were evaluated for tumor necrosis using hematoxylin–eosin staining^18^ T-cells (CD3 +, CD4 +, CD8 +), natural killer cells (CD56 +), vessel density (CD31 +), vascular endothelial growth factor (VEGF), and apoptotic factors (caspase-3) were evaluated by immunohistochemical (IHC) analyses. Immunomodulatory effects were assessed by determining CD3 + (T-cell receptor) (SP7 antibody), CD4 + (T helper cell) (4B12 antibody), CD8 + (cytotoxic T lymphocyte) (4B11 antibody), and CD56 + (natural killer cell) (123C3.D3 antibody) staining^19^. The number of positive cells per high power field (HPF, 400x) were counted only in the vicinity of the tumor nests, averaging over 10 high-power-fields. If less than 1 positive cell per HPF on average was positive, the score was recorded as negative. Mean vessel density (MVD) was assessed by CD31 staining (JC70A antibody)^20^. The number of cells positive per low-power-field (LPF, 100x) was counted. On average, 10 LPFs were included. VEGF (EP1176Y antibody) was assessed by determining the H-score, as defined by (3 × % of intensely-positive tumor cells) + (2 × % of moderately-positive tumor cells) + (% of weakly-positive tumor cells), regardless of magnification^21^. To assess apoptosis (caspase-3) (polyclonal antibody), an H-score was calculated using the same criteria^22,23^.

### Statistical analysis

For continuous variables, means and standard deviations (SD) were used to present normally distributed data, with medians and interquartile ranges for non-normal data. For categorical data, sample size and percentages were computed. For all univariate analyses, Welch’s t-test was used to compare the paired samples and the Wilcoxon rank sum test was used for non-normally distributed data; a two-sided test was used to detect statistically significant differences. A *p*-value of < 0.05 was considered statistically significant. To assess the relationship between dose/volume and H&E necrosis, a loess line was fit to the data to ascertain a linear relationship.

### Conference presentation

Preliminary data for this study were presented at a virtual oral presentation at the Third Annual Conference on Brain Metastases from August 19–20, 2021.

## Results

Twenty-two patients were treated with dose-escalated pre-operative SRS/SRT and resection and had non-irradiated primary tumor samples for comparative analyses. Non-small cell lung cancer (NSCLC) was the most common primary tumor (9/22 patients, 41%) with gynecologic malignancies (4/22, 18%), breast cancer (3/22, 14%), melanoma (2/22, 9%), gastrointestinal (2/22, 9%), and genitourinary (2/22, 9%) representing the remaining cases (Table 1). The median tumor diameter was 3.6 cm (range: 2.2–4.5 cm) and the median gross tumor volume was 14.20 cm^3^ (range: 2.91–31.35 cm^3^). Given the large and symptomatic brain metastases in this series, the majority of patients (15/22, 68%) were on corticosteroids prior to treatment. Most patients (10/22, 45%) received a median preoperative SRS dose of 18 Gy (range: 15–20 Gy) in 1 fraction; 2 patients were treated with pre-operative SRT to a dose of 27 Gy in 3 fractions and 1 patient to 30 Gy in 5 fractions; dose-selection followed pre-defined institutional guidelines. The median duration from SRS/SRT to resection was 67.8 h (range: 18.25–160.61 h); there was a trend toward a shorter interval for those treated with pre-operative SRS versus pre-operative SRT (67.8 h vs. 118.9 h, *p* = 0.06). The median follow-up was 12.3 months and the 1-year freedom from local failure was 95% (95% CI 77–99%).

**Table 1:** Patient and tumor characteristics for those treated with dose-escalated pre-operative stereotactic radiotherapy and surgery.

Patient no	Sex	Age	Primary tumor	BM location	Dexamethasone	Oral/IV	Steroid dose prior Sx (mg)	Interval between SRT to Sx (Hours:Min)	Total dose (Gy)	#Fx	D/Fx	Max linear size (cm)	Tumor volume (cm^3^)	Dose/volume (Gy/cm^3^)
1	F	80	NSCLC	Left Frontal	Yes	IV	18	97.92	15	1	15	4.2	31.35	0.48
2	M	75	Melanoma	Left Frontal	No	No	0	163.15	15	1	15	3.3	15.09	0.99
3	F	71	NSCLC	Left Cerebellar	Yes	IV	10	67.8	15	1	15	3.8	18.43	0.81
4	F	54	Ovary	Right Occipital	Yes	IV	14	67.95	15	1	15	2.9	8.04	1.87
5	M	56	Esophagus	Right Occipital	Yes	IV	10	115.81	15	1	15	3.4	13.91	1.08
6	F	66	Breast	Left Cerebellar	Yes	IV	4	26.47	15	1	15	3.6	17.49	0.86
7	F	68	NSCLC	Right Occipital	Yes	Oral	8	18.25	15	1	15	4.3	28.54	0.53
8	F	75	Ovary	Left Cerebellar	No	No	0	18.25	15	1	15	4.2	8.66	1.73
9	F	35	Breast	Left Parietal	Yes	IV	10	120.21	18	1	18	3.8	15.68	1.15
10	F	64	Ovary	Left Frontal	No	No	0	142	18	1	18	3	10.25	1.76
11	F	35	Breast	Left Frontal	Yes	IV	14	20.77	18	1	18	2.7	7.50	2.40
12	M	59	Bladder	Right Frontal	No	No	0	95	18	1	18	2.7	6.60	2.73
13	F	61	Colon	Left Parietal	No	No	0	63.33	18	1	18	3.0	7.86	2.29
14	M	62	NSCLC	Right Temporal	Yes	IV	4	18.77	18	1	18	3.7	13.45	1.34
15	M	60	NSCLC	Right Cerebellar	Yes	IV	4	22.35	18	1	18	3.8	15.71	1.15
16	F	48	NSCLC	Right Parietal	Yes	IV	12	90	18	1	18	4.5	20.12	0.89
17	F	62	Ovary	Left Parietal	No	No	0	65.43	18	1	18	3.7	15.76	1.14
18	F	60	NSCLC	Left Cerebellar	Yes	IV	12	50.93	18	1	18	3.4	11.43	1.57
19	M	58	NSCLC	Right Parietal	Yes	IV/Oral	8	120.95	20	1	20	2.5	6.96	2.87
20	M	79	Prostate	Left Frontal	Yes	IV	4	118.92	27	3	9	2.2	2.91	1.10
21	F	77	Melanoma	Left Parietal	No	No	0	260.61	27	3	9	4.5	29.55	0.91
22	M	59	NSCLC	Left Frontal	Yes	IV	4	48.6	30	5	6	3.7	14.49	2.07

*IV* intravenous, *Sx* surgery, *SRT* stereotactic radiotherapy, *#Fx* number of fractions, *D/Fx* dose per fraction, *M* male, *F* female, *mg* milligram, *Gy* grey, *min* minute, *cm* centimeter, *cm*^*3*^ cubic centimeter, *NSCLC* non-small cell lung cancer.

**Figure 1:**
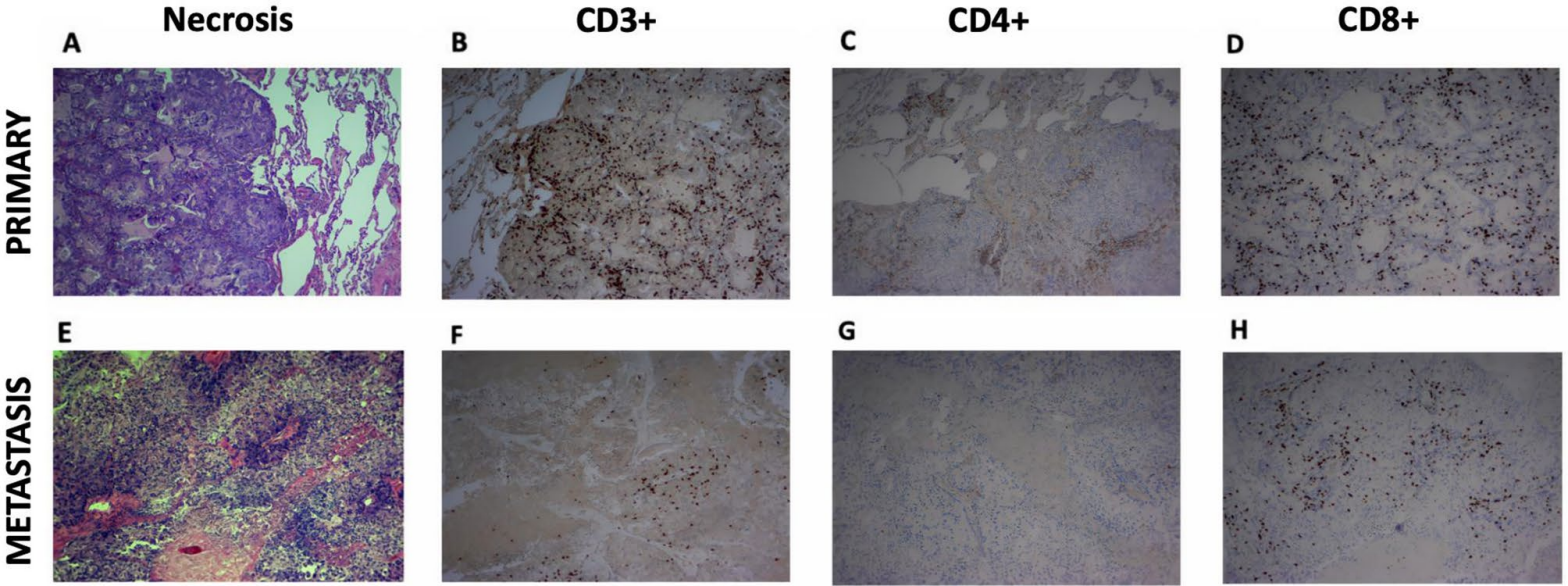
Representative tissue samples displaying the acute effects of stereotactic radiosurgery in brain metastasis. Hematoxylin and eosin staining showing necrosis of a primary tumor (NSCLC) sample (**A**); primary tissue sections were immunohistochemically stained for CD3 + (**B**), CD4 + (**C**), and CD8 + (**D**) cells. Hematoxylin and eosin staining demonstrated an increase in necrosis in the paired resected brain metastasis after pre-operative SRS (**E**). Additionally, a decrease in all immunomodulatory cell populations, including CD3 + (**F**), CD4 + (**G**), and CD8 + cells (**H**) were observed on pairwise comparison. (Original magnification × 40).

**Figure 2:**
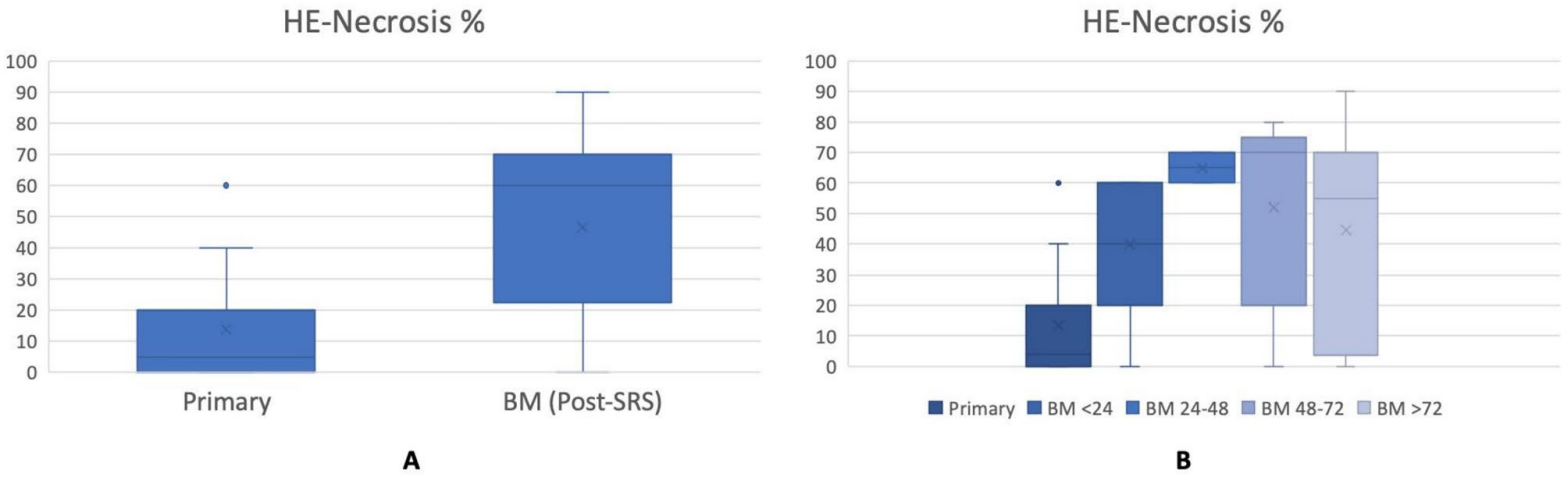
Relationship for the percentage of tumor necrosis between the primary tumor and irradiated BM: (**A**) tumor necrosis in primary tumors was significantly lower than irradiated BM; (**B**) no difference in the proportion of tumor necrosis with respect to time interval from SRT to surgery was observed: primary (non-irradiated), BM < 24 h, 24–48 h, 48–72 h and > 72 h.

Representative tissue samples demonstrating pairwise comparisons of necrosis and immunomodulatory cell populations from a brain metastasis treated with pre-operative SRS and a non-irradiated NSCLC primary tumor are illustrated in Fig. 1. Tumor necrosis was found to be significantly higher in irradiated brain metastases than non-irradiated primary tumor tissues (mean paired difference: 40, SD: 56, *p* < 0.001) (Fig. 2A). There appeared to be no difference in the proportion of tumor necrosis with respect to time interval from SRT to surgery: there was a median of 40% necrosis observed < 24 h after SRT, 65% at 24–48 h, 52% at 48–72 h, and 45% at > 72 h (*p* = 0.56) (Fig. 2B). Given the wide spectrum of tumor sizes in this study, we also looked at the effect of tumor necrosis in patients treated with SRS using a volume-corrected dose analysis (prescribed dose in Gy/tumor volume in cm^3^). No linear relationship was observed on the basis of the loess line. In the three patients (14%) who experienced a local failure, two had cerebellar metastases and the other one was occipital. Interestingly, the necrosis score of the brain metastases that demonstrated local failure was 0 for two patients and only a 10% increase compared to the primary tumor in the third patient.

**Figure 3:**
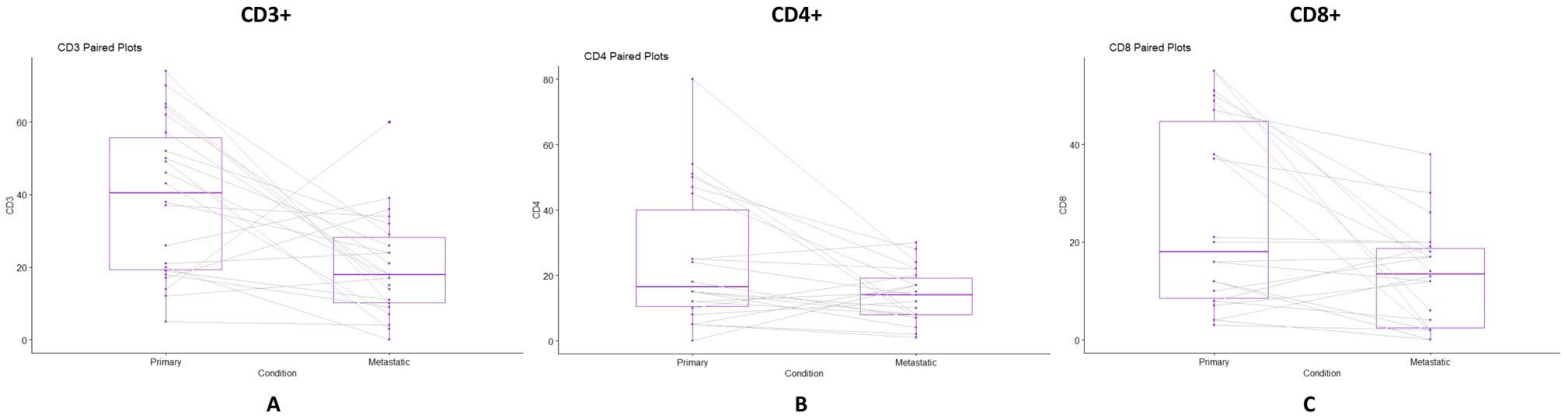
Paired box plots representing the immunomodulatory effects of pre-operative stereotactic radiotherapy on metastasis tumors compared to non-irradiated primary tumors on immunomodulatory cells, including CD3 + (T-cell receptor) (**A**), CD4 + (T helper cell) (**B**), and CD8 + (cytotoxic T lymphocytes) (**C**).

The immunohistochemistry scores of matched primary tumors and brain metastases are presented in Table 2. Overall, pairwise comparisons demonstrated decreases in all immunomodulatory cell populations in irradiated metastases compared to non-irradiated primary tumors, including CD3 + (mean paired difference − 18.5, SD: 26.7, *p* = 0.003), CD4 + (− 10.8, SD: 19.14, *p* = 0.01), and CD8 + cells (− 5.5, SD: 23, *p* = 0.01) (Fig. 3). The use of corticosteroids did not correlate with change in immunomodulatory cell populations (*p* = 0.68) nor did the daily dose of corticosteroid at the time of surgery (*p* = 0.45). There was no dose/volume relationship observed for change in immunomodulatory cell populations (*p* = 0.75). While irradiated brain metastases had numerically lower CD 31 +, CD 56 +, VEGF, and caspase-3 scores than non-irradiated primary tumors, none of the differences were statistically significant (*p* > 0.05). Additionally, time interval between SRS/SRT and surgery had no effect on CD3 +, CD4 + , CD8 +, CD 31 +, CD 56 +, VEGF, or caspase 3 levels.

**Table 2:** Immunohistochemistry scores of the matched non-irradiated primary tumor tissues and brain metastasis treated with pre-operative stereotactic radiotherapy and resection.

Participant	Primary or Mets	Specimen site	CD3	CD4	CD8	CD56	CD31	VEGF	Caspase 3	H&E necrosis %	Time interval	Time interval grouping (h)
1	Primary	NSCLC	52	80	50	0	25	35	80	40	97.92	> 72
	Metastatic	Brain	32	24	26	0	21	3	65	70	97.92	> 72
2	Primary	Melanoma	17	25	21	1	32	7	17	0	163.15	> 72
	Metastatic	Brain	36	22	20	0	45	0	5	0	163.15	> 72
3	Primary	NSCLC	57	51	55	0	32	120	45	60	67.8	48–72
	Metastatic	Brain	18	7	2	0	7	55	15	70	67.8	48–72
4	Primary	Ovary	62	5	47	5	30	0	10	0	67.98	48–72
	Metastatic	Brain	21	17	38	5	24	0	20	40	67.98	48–72
5	Primary	Esophagus	70	15	20	0	32	3	3	5	115.81	> 72
	Metastatic	Brain	29	8	20	0	22	80	80	70	115.81	> 72
6	Primary	Breast	18	0	3	90	23	0	0	0	26.5	24–48
	Metastatic	Brain	9	17	2	70	55	0	7	60	26.5	24–48
7	Primary	NSCLC	26	25	16	0	28	190	20	20	19.75	< 24
	Metastatic	Brain	39	30	12	0	18	200	35	0	19.75	< 24
8	Primary	Ovary	19	24	16	5	22	0	7	0	18.25	< 24
	Metastatic	Brain	11	14	17	5	28	0	3	60	18.25	< 24
9	Primary	Breast	64	47	51	0	17	270	3	0	120.21	> 72
	Metastatic	Brain	14	28	18	12	15	130	15	40	120.21	> 72
10	Primary	Ovary	49	15	38	80	23	65	3	0	142	> 72
	Metastatic	Brain	3	4	2	40	4	30	25	60	142	> 72
11	Primary	Breast	50	45	55	0	35	20	43	0	20.77	< 24
	Metastatic	Brain	26	17	14	1	18	10	65	60	20.77	< 24
12	Primary	Bladder	38	24	38	0	13	225	3	10	95	> 72
	Metastatic	Brain	24	14	17	0	31	270	9	60	95	> 72
13	Primary	Sigmoid Colon	46	12	7	0	52	0	30	0	69.3	48–72
	Metastatic	Brain	18	15	12	0	25	240	50	80	69.3	48–72
14	Primary	NSCLC	37	12	37	20	12	110	16	0	18.77	< 24
	Metastatic	Brain	34	8	30	0	5	110	0	40	18.77	< 24
15	Primary	NSCLC	43	18	12	30	6	120	2	40	22.35	< 24
	Metastatic	Brain	7	7	2	20	8	170	3	40	22.35	< 24
16	Primary	NSCLC	5	5	4	0	4	190	0	0	90	> 72
	Metastatic	Brain	4	2	0	15	10	130	6	50	90	> 72
17	Primary	Ovary	65	50	8	0	27	3	5	20	65.43	48–72
	Metastatic	Brain	15	22	4	40	17	0	3	70	65.43	48–72
18	Primary	NSCLC	74	54	49	0	45	180	1.5	20	50.93	48–72
	Metastatic	Brain	10	8	6	0	15	210	5	0	50.93	48–72
19	Primary	NSCLC	14	15	10	28	15	210	3	60	120.95	> 72
	Metastatic	Brain	60	10	17	0	25	280	23	90	120.95	> 72
20	Primary	Prostate	20	5	12	0	18	0	15	5	118.92	> 72
	Metastatic	Brain	0	1	0	0	25	0	32	0	118.92	> 72
21	Primary	Melanoma	21	10	8	0	24	0	7	3	260.1	> 72
	Metastatic	Brain	24	20	19	0	22	0	5	5	260.1	> 72
22	Primary	NSCLC	12	8	4	0	3	300	70	10	48.6	48–72
	Metastatic	Brain	17	12	13	8	28	230	70	70	48.6	48–72

*CD3* cluster of differentiation 3, *CD4* cluster of differentiation 4, *CD8* cluster of differentiation 8, *CD56* neural cell adhesion molecule, *CD31* platelet/endothelial cell adhesion molecule-1, *VEGF* vascular endothelial growth factor, *Caspase 3* caspase protein 3, *H&E* hematoxylin and eosin, % percentage, *NSCLC* non-small cell lung cancer.

## Discussion

In addition to the classic 4 “R’s of radiation biology” (repair, repopulation, redistribution, and re-oxygenation), the unique effects of radiosurgery commonly confer its designation as the fifth “R”^24^. Yet, published reports evaluating the radiobiologic effects of SRS/SRT are limited to preclinical studies and a few clinical reports in extracranial disease sites, even though treatment of intracranial tumors remains the most common indication for stereotactic treatment. To date, limited data regarding intracranial changes have been published. The present study provides the first in-human comparative evaluation of the acute biologic effects of SRS/SRT employing a comprehensive evaluation of multiple tissue parameters, including tumor necrosis, immunomodulatory cell populations, angiogenesis, apoptosis, and vascular density.

We observed a significantly higher proportion of tumor necrosis in irradiated brain metastases compared to non-irradiated primary tumors. Despite the lack of pre-irradiation tumor biopsies (which may be limited in histologic evaluation and be subject to sampling bias) and given the substantially higher rates of tumor necrosis and the histologies evaluated in this study (which are not commonly associated with innate tumor necrosis), we attributed these effects to SRS/SRT. We previously published indirect, but supportive evidence in a small series of 4 patients evaluated with multiple PET-FDG scans. The results showed significant FDG uptake increase within 24 h, which can occur early in the necrosis/inflammation process^15^. We surmise that tumor necrosis occurs soon (around 24 h after treatment) and that this early effect would persist for an extended window (of a few days) during which resection of the brain metastasis typically occurs. This hypothesis is supported by pre- and post-SRT biopsy specimens from ten patients treated with spine SRT and surgical stabilization where tumor necrosis was not observed in any of the pre-SBRT or same-day surgery biopsy specimens, but was demonstrated in 5 of the 6 biopsy specimens obtained a day after surgery^25^. Although most pre-operative SRT series have utilized a 24 h to one week interval to surgery, and ongoing clinical trials range up to 2 weeks (NCT03750227 and NCT03741673), there is no optimal time window suggested by this collective analysis.

Dose and fractionation schedules for treatment of brain metastasis are based on the empirical evidence balancing the rate of local disease control with the risk of radiation necrosis^26^. These decisions are based on imaging-defined definitions without comparative histologic analyses. Pre-operative radiosurgery series typically utilize a 20% dose-reduction strategy, with multiple institutions using doses as low as 12 Gy in 1 fraction^12^. In our series we employed an approach utilizing the definitive doses for intact brain metastases established in cooperative group trials^27^ and validated in a recently conducted phase I dose-escalation trial in patients undergoing pre-operative SRS and resection^16^. Although systematic dose-escalation SRT experiences with extracranial tumor sites, such as prostate cancer, have demonstrated a dose-dependent increase in tissue effects (albeit with 2-year post-treatment biopsies) which correlate with risk of local failure^28^ a similar relationship was not observed in this study. However, our post-SRS/SRT tissue acquisition window was very narrow. The 1-year actuarial local control rate of 95% achieved with higher-than-conventional dosing is promising and merits long-term follow-up and study. Of note, it is interesting that descriptively, two of the three patients with a local failure had 0% necrosis in the resected tumor sample, and the third patient with only a 10% higher necrosis rate than the primary tumor. In our prior PET study, failure to respond with acute FDG uptake elevation was associated with local failure^15^ leading us to postulate that the two phenomena could be correlated. Therefore, this study also supports the safety of SRS dose-escalation in this phase 1 trial, which can be further explored in future clinical trials, where it would be very useful to correlate dose with both tissue changes and clinical outcomes.

The effect of SRS/SRT on the immunomodulatory cell populations was an interesting finding of this study. In comparison to primary tumors, we observed a decrease in populations of CD3 +, CD4 +, and CD8 + cells in irradiated and resected brain metastases. Although we did not observe a dependency of these factors on dose or time interval from SRT to surgery, this finding is tempered by the small sample size and short study window. The minimal fractional dose of 7–8 Gy known to induce T-cell infiltration, primarily via CD8 + cells^29^, was exceeded in all but one patient in this series (treated at 6 Gy per fraction). However, very high fractional doses, such as those used in this series exceeding 15–18 Gy in 1 fraction, may actually induce T-regulatory cell activity and downregulate the immunomodulatory effects of radiotherapy^30^. Moreover, although necrosis is commonly associated with inflammation, the finding of lower immunomodulatory cell populations in irradiated metastases may be due to the short interval from SRT to surgery, prior to immunomodulatory cell infiltration^31^. In addition to these radiotherapy-related effects, we observed no association between corticosteroid use/dose and changes in immunomodulatory cell populations. As an increasing proportion of patients receive immunotherapeutics for treatment of their metastatic disease, an in-depth analysis of pre-operative radiosurgery specimens to systematically study changes as a result of dose and fractionation may further inform clinical practice, especially if there is any association between change in cell populations and the efficacy of these agents.

A few studies in a variety of extracranial disease sites have also evaluated the tissue effects of SRS/SRT. A phase I trial of pre-operative SRT for prostate cancer (25 Gy in 5 fractions), followed by radical prostatectomy two weeks later, demonstrated a lack of change in apoptosis in the resected tissue samples but did demonstrate reduced cell proliferation measured by p21 WAF activation^32^. A phase I trial of pre-operative partial breast irradiation (15, 18, and 21 Gy in 1 fraction), followed by lumpectomy within 10 days, not only revealed gene expression changes in post-irradiated tumor samples compared to pre-irradiation biopsies, but also demonstrated dose–response effects in parameters related to immunity and inflammation^33^. A study of pre-operative SRS (20 Gy in 1 fraction) for breast cancer, followed by breast-conserving surgery three months later, demonstrated a median residual tumor cellularity of only 3% in 8/10 patients^34^. A phase II trial of pre-operative SRT (54–60 Gy in 3–8 fractions) for patients with early-stage NSCLC, followed by lobectomy or sublobar resection, demonstrated a pathologic complete response rate of 60% via H&E staining but did not report other tissue parameters, such as necrosis or apoptosis^35^. Unlike these series, pre-operative SRS/SRT in our study was not associated with changes in other tissue parameters including vessel density, apoptotic factors, or VEGF. However, it is important to note that these other studies compared pre- and post-radiotherapy biopsy specimens of the treated tumors, not irradiated metastases compared to non-irradiated primary tumors. Other factors that may account for these differences include the short window between SRS/SRT and surgery in our series compared to the interval between radiotherapy and surgery in extracranial sites^25,36^, differences in tumor histologies represented, and differences in the response to radiotherapy in intracranial sites compared to extracranial sites given the inherent differences in the tumor microenvironment^37^.

This study has several limitations. First, although eight different primary tumor histologies were represented in this study, the majority of patients (41%) had NSCLC. As tumor microenvironments are related to primary tumor histology and molecular profile, this prevents extrapolation of the results of this study to other histologies not well represented as all histologies are distinct and underrepresented in this limited series. For example, certain histologies, such as renal cell carcinoma, melanoma, and thyroid cancer have innate tumor hemorrhage and necrosis, which would be difficult to differentiate from the effects of radiotherapy^38^. Further, although the majority of our patients had NSCLC, even within this entity, there are several molecular subtypes, and hence one cannot generalize these results to all tumor types and subtypes. Moreover, given that the tumor histology and molecular profile ultimately dictate systemic therapy, the impact of this on the tumor microenvironment could not be evaluated in a study with this small sample size. Second, we compared irradiated brain metastases to non-irradiated primary tumor samples, given the lack of ability to obtain pre-radiotherapy biopsies. Differences in the tissue samples between these sites are known to exist, although these are typically related to genomic drivers^39^ or receptor subtypes^40^. Third, although a number of tissue parameters were evaluated, the sensitivity of different methods of analysis, for example differential gene expression via RNA-sequencing^41^, may yield additional insight above that detected via IHC analyses alone. Fourth, the overall sample size of this study is small and therefore limits definitive conclusions regarding the effect of SRS on brain metastases. Further, this limits the power of the study to evaluate the impact of dose, tumor histology, systemic therapy, and timing of SRS on these tissue parameters. Therefore, current studies in which patients are randomized to pre-operative versus post-operative SRS (i.e. NCT03750227 and NCT03741673) and future studies (i.e. recently activated NRG BN012) could provide valuable opportunities to further elucidate this question through collection of both primary tumors and extracranial metastases in addition to intracranial metastases.

## Conclusion

In this series, dose-escalated pre-operative SRS/SRT was associated with favorable rates of tumor control, and as compared to the primary non-irradiated tumor, increased tumor necrosis and a reduction in multiple immunomodulatory cell populations. Differences in immunomodulatory factors may be consequential to multiple factors, including corticosteroid use and the immunosuppressive effect of high-dose SRS/SRT. Understanding this complex interplay in a larger sample size is critical for a better understanding of the impact of SRS/SRT in the brain.
